# Supplementary material for: Epigenetic Distribution of Recombinant Plant Chromosome Fragments in a Human–Arabidopsis Hybrid Cell Line
Source: Int J Mol Sci. 2021 May 21;22(11):5426. doi: 10.3390/ijms22115426 (PMC8196797; doi:10.3390/ijms22115426)
Supplement: Supplementary file 1 [file ijms-22-05426-s001.zip › ijms-1185504-SI.pdf]

Article

Epigenetic Landscape of Recombinant Plant Chromosome Fragments in a Human-*Arabidopsis* Hybrid Cell Line

Yeng Mun Liaw[1,§], Yikun Liu[1,§], Chee How Teo[2], Petr Cápal[3], Naoki Wada[4], Kiichi Fukui[5], Jaroslav Doležel[3] and Nobuko Ohmido[1*]

[1]Graduate School of Human Development and Environment, Kobe University, Kobe 657-8501, Hyogo, Japan; liawlym@gmail.com

[2]Centre for Research in Biotechnology for Agriculture, Universiti Malaya, 50603 Lembah Pantai, Kuala Lumpur, Malaysia; cheehow.teo@um.edu.my

[3]Institute of Experimental Botany of the Czech Academy of Sciences, Centre of the Region Hana for Biotechnological and Agricultural Research, Šlechtitelů 31, 779 00 Olomouc, Czech Republic; capal@ueb.cas.cz; dolezel@ueb.cas.cz

[4]Graduate School of Technology, Industrial and Social Sciences, Tokushima University, Tokushima 770-8503, Tokushima, Japan; wada.naoki@tokushima-u.ac.jp

[5]Graduate School of Pharmaceutical Sciences, Osaka University, Suita 565-0871, Osaka, Japan; kfukui@bio.eng.osaka-u.ac.jp

* Correspondence: ohmido@kobe-u.ac.jp; Tel: 81-78-803-7763

§ These authors contributed equally to this work

Table S1. Methylation profiles of 300-day-old hybrid cell obtained using BSMAP and Bismark

	BSMAP	Bismark
CG	23.8 %	23.8 %
CHG	0.27 %	0.3 %
CHH	0.26 %	0.3 %

Table S2. Number of occurrences of overlapping repeat families in hypomethylated regions overlapping with TAIR UCSC repeatmasker tracks

Satellites		LTR	
Repeat families	Overlapping occurrence	Repeat families	Overlapping occurrence *
COLAR12	193	Athila4B_LTR	101
ATSAT5	37	Athila2_LTR	83
ATSAA5	27	Athila6A_LTR	76
AR12	14	Athila4C_I-int	63
ATSAB5	13	Athila_I-int	61
ATCLUST1	6	ATHILA6C_I-int	61
ATENSAT1	2	Athila4_I-int	54
ATENSAT2	1	Atlantys1_I-int	52
		Athila2_I-int	52
		Athila3_I-int	51
		Athila6A_I-int	50
		Athila4D_LTR	50
		ATGP10_I-int	47
		Athila_LTR	46
		Athila3_LTR	46
		Athila4A_I-int	44
		Atlantys2_I-int	43
		Athila6B_I-int	42
		Athila0_I-int	40

*Only an occurrence of at least 40 is shown here.

Table S3. Sequences of the primers used for expression analysis

Gene name	Primer sequences (F: Forward, R: Reverse)	Reference
DDM2	F: GCGGTTCTCAGTCACACGATCC<br>R: GGGGCATAAAGTTTTGATGGTTTCC	
DRM2	F: GTGCTGCTCAAATGGCTAGGGAAT<br>R: GCTCGTTTGGAACCCCAAATCCTAT	
GSL8	F: TGGGGTTTTCCCTGAAGTACGTG<br>R: GGGACTGTGCATTTGAAAGTGTGAG	Han, S., & Kim, D. (2006)
CESA4	F: AGATGCGGAGTGGAAAGAACGTGTA<br>R: ACGATGCGGTAAGGACTGATTTTGC	Han, S., & Kim, D. (2006)
HPR	F: CGCTTGCAGCTCTCAACGTC<br>R: GGCGGTGAAGCGTTTTCGTT	
NPY4	F: CTAGCTTCCCAAGACACTCTCAC<br>R: GCCCGCATACTCTCTTCTTCTC	
LHCB	F: GGCCATGCTTGGCACTCTA<br>R: GTCAACGAGAACGGCAATGGT	
CA2	F: GTCCCTCTCATCGCCTGTCTC<br>R: AGCTTCGATGGCGTCTTCAT	
APE2	F: CTCCGGTCGTCAGCTTCGT<br>R: GCATCCCCAGCGGTATCTCC	
SBE2.2	F: TATAGAGAGTGGGCGCCTGG<br>R: ACCATCAGTGTTGTTGGGCA	
AtNADH	F: CCCTCTCCTCCTCCAATCTC<br>R: ACCTCCATTGGCACAACTTC	Wada et al (2017)
TIP	F: AAAAATCATCAAATCCATCTCCCAC<br>R: GGCAAAAAGAGATATTGCAACACTTG	Wada et al (2017)
PLP6	F: TGGAGCAGCTGATACTGTGG<br>R: TTGCTTGAACGCTACAGTGG	Wada et al (2017)
